# A structural equation model in adults with type 1 and 2 diabetes: exploring the interplay of psychological states and diabetes outcomes, and the mediating effect of resilience

**DOI:** 10.1007/s00592-022-01955-3

**Published:** 2022-08-30

**Authors:** Rosalind Pate, Noreen Caswell, Kathryn Jane Gardner, Lynda Holyoak

**Affiliations:** School of Psychology, University of Central Lancashire, Preston, UK

**Keywords:** Structural equation model, Diabetes mellitus, Resilience, Anxiety, Diabetes distress, Cognition

## Abstract

**Aims:**

Type 1 and 2 diabetes mellitus (T1DM and T2DM) can lead to emotional distress and cognitive impairments, often caused by psychological factors such as low mood or anxiety; yet, few studies have explored the theoretical mechanisms underlying these relationships and within one study. This study explored the relationships between psychological states (anxiety/worry, fatigue) and diabetes outcomes (diabetes distress, cognitive dysfunction), and whether resilience mediated the association between these in T1DM and T2DM.

**Methods:**

A sample of 307 UK adults with a clinical diagnosis of diabetes (T1DM = 129; T2DM = 178) completed a cross-sectional online survey, composed of six questionnaires. Associations between variables were investigated using Pearson’s correlations and Structural Equation Modelling (SEM).

**Results:**

Psychological states were significantly correlated with diabetes outcomes, and resilience was significantly related to both psychological states and diabetes outcomes. The SEM model achieved an acceptable model fit with a significant mediating effect of resilience between psychological states (anxiety/worry, fatigue) and diabetes outcomes (diabetes distress, cognitive dysfunction), with no significant differences between diabetes type.

**Conclusions:**

We propose a new theoretical model of T1DM and T2DM that could be used to provide guidance for those designing interventions. These findings help to understand the complex nature of diabetes management, suggesting resilience could be a key factor in managing psychological states and diabetes outcomes.

## Background

The two main classifications of diabetes are T1DM and T2DM, and whilst they share clinically similar symptoms, the aetiology of the disorder types differs significantly [1]. Both have a strong genetic component [1], and inadequate long-term self-management is associated with greater risk of serious acute complications (e.g., coma) and chronic (e.g., cardiopathy, sexual dysfunction, retinopathy, nephropathy, limb loss) [2]. Approximately one third of people with T2DM and two thirds of people with T1DM do not achieve the target glycated haemoglobin (HbA1c) levels [2]. The constant demands of living with diabetes can also take a significant psychological toll, with many individuals experiencing distress, depressed mood, anxiety, fatigue and reduced quality of life [3, 4]. The rising burden of diabetes globally is a major health priority, placing increased demands on patients, carers, health systems and society [5]. Identifying and understanding the key psychological factors that contribute to diabetes management and outcomes is therefore a key priority.

**Fig. 1:**
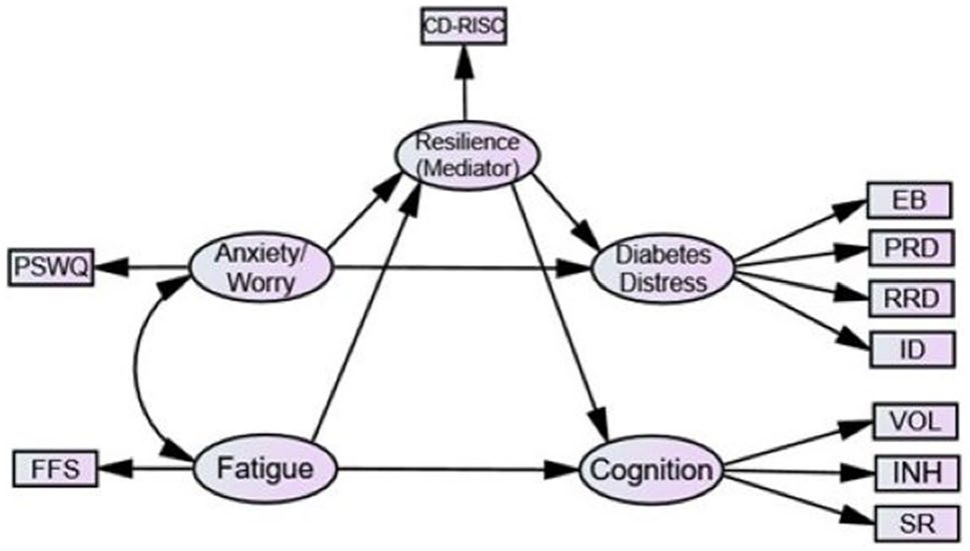
Proposed Structural Model: Psychological States (Anxiety/Worry, Fatigue), and Diabetes Outcomes (Diabetes Distress, Cognition), with Resilience as Mediator. *Key:* PSWQ (Penn State Worry Questionnaire); FFS (Flinder’s Fatigue Scale); CD−RISC (Connor-Davidson Resilience Scale), DDS (Diabetes Distress Scale, using four subscales: Emotional Burden, Physician Related Distress, Regimen Related Distress, Interpersonal Distress); DEX (Dysexecutive Questionnaire, using three subscales: Volition, Inhibition and Social Regulation). The proposed structural model shows association pathways between predictor variables (anxiety, fatigue) and diabetes outcome variables (diabetes distress, cognition), mediated by resilience. The boxes indicate the measures used to assess their respective variable

Existing literature shows both T1 and T2 diabetes suffer from high levels of anxiety and fatigue, with diabetes distress and executive functioning (EF) issues [8, 9]. Diabetes distress and EF issues are positively associated with each other, and with anxiety and fatigue [8–19]. Resilience is an increasingly important factor in diabetes self-management because resilience can be taught through intervention [27, 45]. Resilience is defined as the capacity to adapt and maintain psychological and physical ‘wellbeing’ in the face of adversity [6] and has been found to correlate negatively with anxiety, fatigue, diabetes distress and cognitive dysfunction [6, 7, 20–28]. These patterns of associations suggest that resilience may act as a mediator [29] but not a moderator; research suggests a causal relationship between anxiety/fatigue and resilience, and resilience and diabetes distress/cognition, therefore, resilience cannot theoretically be a moderator variable [29]. Studies have yet to explore the role of resilience in conjunction with anxiety, fatigue, diabetes distress and executive dysfunction simultaneously, within one model; this is the focus of the present research and is depicted in Fig. 1.

Existing literature suggests direct associations between anxiety (predictor) and diabetes distress and cognition (outcomes), and between fatigue and cognition. There is still little research to suggest a direct link between fatigue and diabetes distress [8]. Park et al. [30] found the relationship between fatigue and diabetes distress was mediated by diabetes symptoms, suggesting the relationship may be an indirect one, and is reflected as such in the proposed model (see Fig. 1).

Winkley et al. [31] suggest future research should focus on underlying theories, rather than replicating existing psychological models that usually deliver small effect sizes. Since theory-based interventions are more likely to produce longer-lasting and larger effects than those without [32], this supports the need to focus more on the underlying theoretical mechanisms, with a focus on the psychosocial effects of living with and managing diabetes [3].

Therefore, the overall aim was to formulate and test an original theoretical model, based on a critical review of existing literatures, that could be used to provide guidance for those designing interventions for T1DM and T2DM groups. More specifically, the study aims were to: 1) to explore whether resilience mediated the association between psychological states and diabetes outcomes, and 2) to assess whether T1DM and T2DM diabetics differ in relation to covariances amongst the afore-mentioned variables.

Based on the above literature, the hypotheses for the proposed structural model are as follows:

Direct relationships will be:

- **H**_**1**_: Anxiety/worry will be positively related to diabetes distress [14–16]
- **H**_**2**_: Anxiety/worry will be negatively related to cognition [17–19, 22]
- **H**_**3**_: Fatigue will be negatively related to cognition [9, 12, 13, 15]

The mediating relationships will be:

- **H**_**4, 5, 6,7**_: Anxiety and fatigue will predict diabetes outcomes of distress and cognition, and these relationships will be mediated by resilience [6, 7, 20–28]

## Method

### Design

A correlation design was used to investigate the strength and direction of associations between the following variables in people with T1DM and T2DM: anxiety, fatigue, diabetes distress, cognition and resilience (as a potential mediator).

### Participants

Adults in the Northwest UK with T1DM (*n* = 129) and T2DM (*n* = 178) were recruited via diabetes support groups, local newspaper advertisements and social media advertisements. Participants completed an e-survey, created using the platform Qualtrics. Exclusion criteria included people below the age of 18 years, those with diabetes that is not T1DM/T2DM, and those who failed to state diabetes type.

Both T1DM and T2DM groups were mostly female (77.5 and 79.2%, respectively), with mean ages of 432.41 (*SD* = 178.99*)* and 663.99 (*SD* = 129.65*),* and were white British (80.6% and 72.5%, respectively). T1DM durations ranged from 5—960 months (80 years), whereas T2DM ranged from 1 to 444 months (37 years). Average diabetes durations were 235.71 (T1DM), 97.08 (T2DM), and average HbA1c levels were 62.11 mmol/mol (T1DM) and 62.271 mmol/mol (T2DM).

### Sample size requirements for structural equation modelling

Guidance taken from Boateng [34] and Wolf et al. [35] was used to inform sample size; sample sizes of 100–200 are recommended, or at least 5 cases per model parameter. See also [35, 36] for SEM discussion with smaller sample sizes.

## Materials and procedure

Participants were provided a link to an anonymous survey, first directing them to the study participation information sheet. Consent was confirmed through completing and submitting the questionnaires. Any participants who did not submit at the end of the survey were deemed to have withdrawn and their data was not used.

### Anxiety/worry (psychological state latent variable)

Anxiety/worry was assessed using the Penn State Worry Questionnaire (PSWQ) [48], a 16-item single factor scale that is considered the ‘gold standard’ for measuring unspecific worry. Scores range between 16 and 80, with a higher score indicating higher anxiety. Internal consistency (Cronbach’s alpha) for this sample was 0.97 and 0.98 for T1DM and T2DM groups, respectively.

### Fatigue (psychological state latent variable)

Fatigue was measured using Flinder’s Fatigue Scale (FFS) [49], a 7-item single factor scale measuring daytime fatigue (e.g., frequency, severity), often associated with insomnia. Six items use a 5-point Likert scale, and item 5 uses a multiple item checklist. Scores range between 0–31, with higher scores indicating greater fatigue. Internal consistency for this sample was 0.92 and 0.90 for T1DM and T2DM groups, respectively.

### Diabetes distress (diabetes outcome latent variable)

Diabetes-specific distress was assessed using the Diabetes Distress Scale (DDS) [50], a 17-item, 4-factor measure consisting of emotional, physician, regimen and interpersonal distress subscales. (Overall and subscale scores were used in correlational analyses, but only subscales were used in the SEM.) Scores range between 17 and 102, where higher scores indicate greater distress. Items relating to their respective factor are averaged, where a mean score of 3 or higher indicates moderate distress, worthy of clinical attention. Internal consistency for this sample was 0.94 and 0.95 for T1DM and T2DM groups, respectively.

### Cognition (diabetes outcome latent variable)

Cognition/EF was assessed using the Dysexecutive Questionnaire (DEX) [51], a 20-item, three-factor measure consisting of volition, inhibition and social regulation subscales. Scores range between 0 and 80, with higher scores indicating greater problems with EF. Internal consistency for this sample was 0.88 for T1DM and T2DM groups.

### Resilience (mediator variable)

Resilience was measured using the Connor-Davidson Resilience Scale (CD-RISC) [52], a 25-item single factor scale, where resilience is defined as the capacity to adapt and maintain psychological and physical wellbeing in the face of stress, adversity or trauma [6]. Scores range between 0–100, where higher scores indicate greater resilience. Internal consistency for this sample was 0.95 for T1DM and T2DM groups.

## Data analytic strategy (SEM)

Structural equation modelling was chosen for analysis as it is a flexible method that can be used to explore relationships between latent variables [34], using AMOS (version 27). Two psychological state latent variables (anxiety and fatigue) were categorised as predictor variables. A third latent variable was resilience (mediator variable). Two diabetes outcome latent variables (diabetes distress and cognition) were categorised as outcome variables. Figure 1 shows the operationalisation of exogenous, endogenous and mediator variables. As recommended in SEM literature [34], an alternative model was also tested, whereby diabetes distress was incorporated as an exogenous variable rather than endogenous, based on existing literature findings [30].

The maximum likelihood chi-square statistic was used to evaluate the measurement and structural models, but as this is sensitive to sample size, the normed chi-square (χ^2^ /*df*) was also used alongside several other indices to assess model fit, including: comparative fit index (CFI), Tucker-Lewis index (TLI), incremental fit index (IFI), root mean square error of approximation (RMSEA) and standardised root mean square residual (SRMR). Hu & Bentler [37] suggested acceptable cut-off criteria indicative of good model fit are > 0.95 (CFI, TLI, IFI). However, given the exploratory nature of this study, it was decided best to use more conservative criteria, indicative of moderate fit. Therefore, the following cut-offs for acceptable model fit were used: χ^2^/*df* 1–5; CFI, TLI and IFI > 0.90; RMSEA ≤ 0.06—0.08; and SRMR ≤ 0.08 [37, 38]. Standardised regression weights were used to interpret direct effects, and bias-corrected bootstrap confidence intervals technique was used to assess the significance of standardised indirect effects.

## Results

### Preliminary analyses

Data were examined for missing values, outliers and normal distribution. Missing value analysis for validated measures were < 5%, missing variables at random (Little’s MCAR test: Chi-Square = 42,895.414, *df* = 42,606.00, *p* = 0.161). Missing values were replaced using person mean substitution by scale/subscale. Outlier detection revealed seven univariate outliers that were dealt with using winzorising, and five multivariate outliers were removed. Given the large sample size (*n* > 300), distribution shape/skewness and kurtosis were used to determine normality. Mean and *SD* values were calculated for all variables recorded, separately for T1 and T2 groups, and independent samples t tests, Pearson’s correlations and SEM analyses were then run separately for each diabetes group.

### Correlational analyses

Pearson’s correlations were conducted to examine **a)** the relationship between psychological states (anxiety, fatigue) and diabetes outcome measures (diabetes distress, cognition), **b)** multicollinearity i.e., high correlations between indicators of psychological state latents and indicators of the outcome measures with each other and **c)** the potential for a mediating relationship of resilience between psychological states and diabetes outcomes. These correlations were undertaken for T1DM (*n* = 129) and T2DM (*n* = 178) diabetes groups separately. Effect sizes (*r* values) were deemed to be small, medium or large if they were, 0.10, 0.30 or 0.50, respectively [39].

T1DM Correlations: All psychological state and diabetes outcome variables were significantly positively correlated, except from the cognition (social regulation only) values. Correlations between components of diabetes distress, anxiety, fatigue and cognition were significant and typically moderate in strength. All psychological state and diabetes outcome variables were significantly negatively associated with resilience values, and typically moderate to strong in strength.

T2DM Correlations: All psychological state and diabetes outcome variables were significantly positively correlated, except from the cognition (social regulation only) values, as in T1DM. Correlations between components of diabetes distress, anxiety, fatigue and cognition were significant and typically moderate in strength. All psychological state and diabetes outcome variables were significantly negatively correlated with resilience values, and typically moderate to strong in strength.

## Measurement models: confirmatory factor analysis (CFA)

### Factor loading, and reliability and validity

Using a factor loading of at least 0.40 [34], 6 items were deleted (see Appendix 3). To assess the reliability and validity of scales used, Composite Reliability (CR) and Average Variance Extracted (AVE) values were calculated, and all scales met acceptable cut-off values for Cronbach’s alpha (> 0.70) and CR (> 0.60), demonstrating adequate reliability [40].

### Multigroup CFA: model fit statistics, T1DM and T2DM groups

Pearson’s correlations were conducted between the latent variables in both diabetes groups; all correlations between psychological states, resilience and diabetes outcomes were significant, with no evidence of multicollinearity (*r* ≤ 0.90).

Fit indices are presented in Table 1; all indicators loaded onto their respective factors (> 0.40), and each scale achieved acceptable values in ≥ 3 fit indices (see Table 1). This suggests the data are suitable for SEM analysis.

**Table 1:** Confirmatory Factor Analysis Fit Indices (Chi Square, CFI, IFI, TLI, RMSEA and SRMR) in T1DM and T2DM Groups

Scale	*χ*^2^/*df *(≥ 1 to 5)	Comparative Fit Index (CFI; ≥ 0.90)	Incremental Fit Index (IFI; ≥ 0.90)	Tucker-Lewis Index (TLI; ≥ 0.90)	Root Mean Square Error of Approx. (RMSEA; ≤ 0.06—0.08)	Stand. Root Mean Square Residual (SRMR; ≤ 0.08)
DDS	2.33 (*p* < 0.001)	**0.93**	**0.93**	**0.91**	**0.07**	0.16
PSWQ	4.06 (*p* < 0.001)	**0.90**	**0.91**	0.89	0.10	**0.07**
FFS	2.50 (*p* < 0.001)	**0.98**	**0.98**	**0.97**	**0.07**	**0.06**
DEX	1.88 (*p* < 0.001)	**0.94**	**0.94**	**0.92**	**0.05**	**0.07**
CD-RISC	2.15 (*p* < 0.001)	0.89	0.89	0.88	**0.06**	**0.07**

Key: *Psychological States:* PSWQ (Penn State Worry Questionnaire); FFS (Flinder’s Fatigue Scale). *Resilience:* CD−RISC (Connor−Davidson Resilience Scale). *Diabetes Outcomes:* DDS (Diabetes Distress Scale); DEX. (Dysexecutive Questionnaire)

### Structural models

**Fig. 2:**
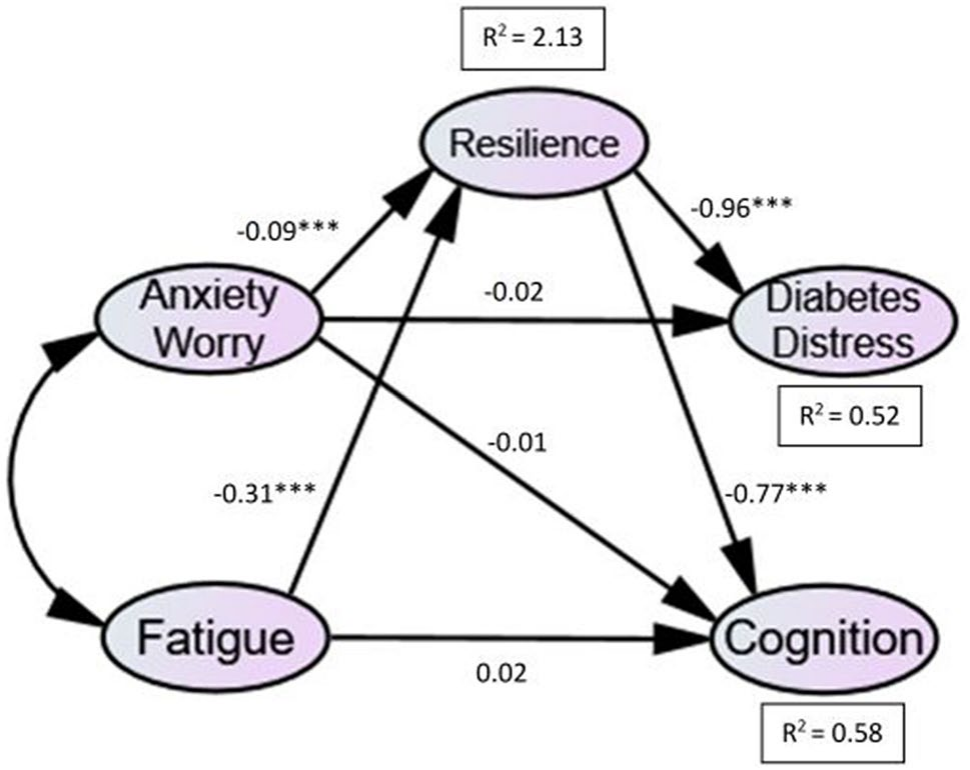
Structural Model for T1DM. The structural model shows pathway coefficients between predictor variables, resilience and diabetes outcome variables in the T1DM group. All indirect pathways (i.e. involving the mediator) were significant, and direct pathways were non-significant) *** *p* =  < 0.001, ** p =  < *0.05*

**Fig. 3:**
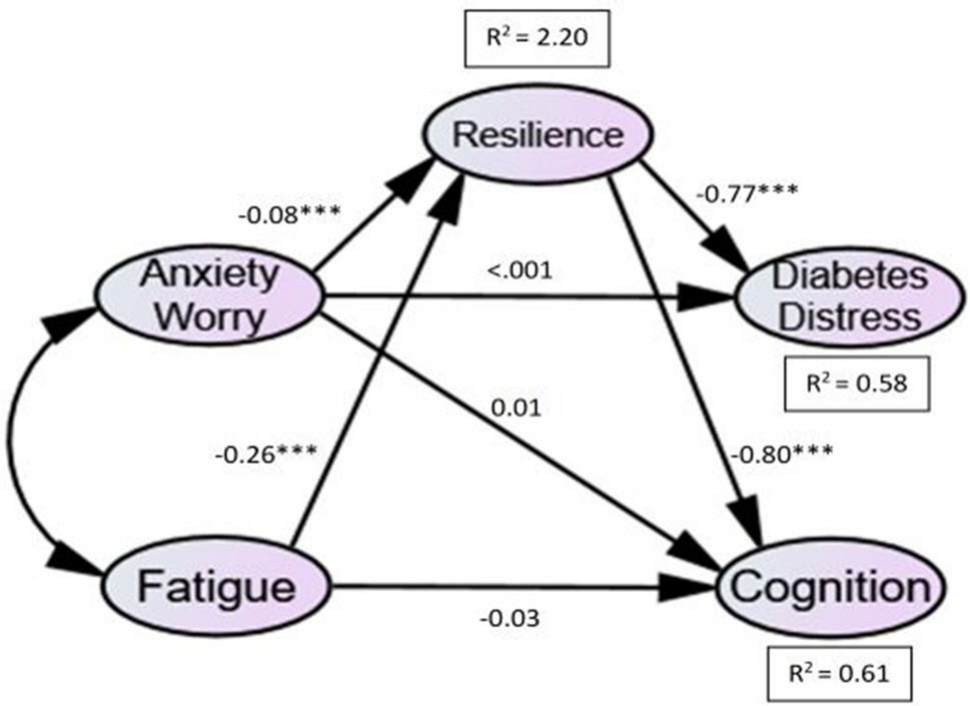
Structural Model for T2DM. The structural model shows pathway coefficients between predictor variables, resilience and diabetes outcome variables in the T2DM group. All indirect pathways (i.e. involving the mediator) were significant, and direct pathways were non-significant) *** *p* =  < 0.001, ** p =  < *0.05*

A diagrammatic representation of the structural model (for T1DM and T2DM) is presented in Figs. 2 and 3. This shows the standardised path coefficients, significance levels and R_2_ values, which indicate the amount of variance explained by the independent variables. The values of fit statistics for the structural model were all found to be within acceptable limits: *χ*^2^ (Chi-Square) = 139.905, *df* = 58, and *χ*^2^/*df* ratio = 2.41, CFI = 0.94, IFI = 0.94, TLI = 0.90, RMSEA = 0.07, and SRMR = 0.07.

The results of the initial hypotheses tests for each path in the structural model are summarised in Table 2.

**Table 2:** SEM results for T1DM and T2DM groups, showing standardised coefficients, *t*-values and significance

Hypothesised path	Standardised coefficients, β	*t* (C.R.)	*p*	Hypothesis
*Direct relationships*				
H_1_ Anxiety/Worry → Diabetes Distress	−0.02 **(0.01)**	−1.59 **(−0.07)**	0.112 **(0.942)**	Not Supported
H_2_ Anxiety/Worry → Cognition	−0.01 **(0.01)**	−0.90 **(0.98)**	0.369 **(0.325)**	Not Supported
H_3_ Fatigue → Cognition	0.02 **(−0.03)**	0.64 **(−1.00)**	0.525 **(0.317)**	Not supported
*Paths in the indirect/ mediating effect*				
H_4_ Anxiety/worry → Resilience H_5_ Fatigue → Resilience	−0.09 **(−0.08)** −0.31 **(−0.26)**	−5.21 **(−6.18)** −4.12 **(−5.85)**	< 0.001 **(< 0.001)** < 0.001 **(< 0.001)**	Supported Supported
H_6_ Resilience → Diabetes Distress	−0.96 **(−0.77)**	−6.26 **(−6.56)**	< 0.001 **(< 0.001)**	Supported
H_7_ Resilience → Cognition	−0.77 **(−0.80)**	−5.21 **(−5.79)**	< 0.001 **(< 0.001)**	Supported

T1DM: Chi−Square = 139.905, df = 58, *p*<.001, *n*=129; T2DM: Chi−Square = 139.905, df = 58, ***p***<.001, ***n***=178 (T2DM values are formatted in bold with brackets)

#### SEM: T1DM

Findings were similar for both T1DM and T2DM. As shown in Table 2, hypotheses 1, 2 and 3 were non-significant and therefore unsupported. However, hypotheses 4, 5, 6 and 7, which are part of the indirect effect were all supported: anxiety and fatigue had a significant negative influence on resilience, and resilience had a significant negative influence on diabetes distress and cognition.

To identify the presence of mediation, bootstrapping was used to calculate direct and indirect effects in T1DM. Results confirmed a mediating effect of resilience on the relationships between psychological states (anxiety, fatigue) and diabetes outcomes (diabetes distress, cognition) in T1DM.

#### SEM: T2DM

For T2DM, hypotheses 1, 2 and 3 were non-significant and therefore unsupported. However, hypotheses 4, 5, 6 and 7 were all supported: anxiety and fatigue were found to have a significant negative influence on resilience, and resilience has a significant negative influence on diabetes distress and cognition.

To confirm the presence of mediation, bootstrapping was used to calculate direct and indirect effects in T2DM. Results confirmed a mediating effect of resilience on the relationships between psychological states (anxiety, fatigue) and diabetes outcomes (diabetes distress, cognition) in T2DM.

### Model comparisons

The above SEM model was used as a baseline comparison, and non-significant pathways were consecutively constrained to zero to confirm whether eliminating non-significant pathways results in a more parsimonious final model [34]. The paths of H_1-3_ were consecutively constrained in models 1, 2 and 3, after which a full mediation model was tested in model 4; all direct paths from psychological states to diabetes outcomes were constrained to zero, leaving only indirect paths (see Appendix 6 for comparison table).

**Fig. 4:**
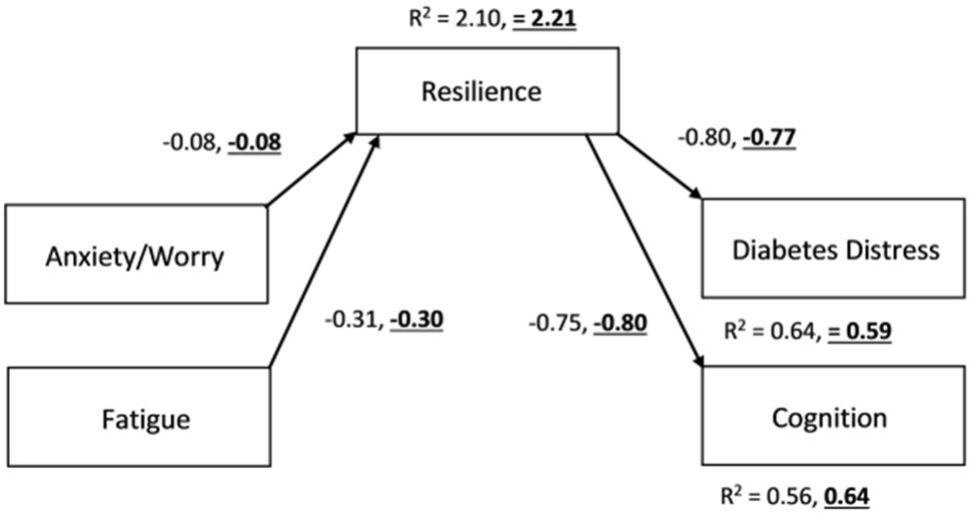
Final Structural Model for T1DM and T2DM. This shows the most parsimonious model, with all non-significant paths removed. All indirect pathways (i.e., involving the mediator) were significant at the *p* < 0.001 level. Standardised beta coefficients are provided for each pathway, indicating effect strength and direction of each predictor variable on outcome variable, and R_2_ values indicate the amount of variance explained by the independent variables (T2DM values are formatted in bold, underlined)

Model 4 is the most parsimonious solution; the non-significant pathways have been eliminated without negatively impacting the model fit statistics, and therefore, model 4 was used as the final structural model (see Fig. 4). Multigroup analysis of Model 4 revealed no significant differences between T1DM and T2DM groups (χ^2^= 8.68, *p* = 0.730), suggesting the model is appropriate to both groups.

In summary, structural equation modelling identified a significant mediational effect of resilience on psychological states (anxiety, fatigue) and diabetes outcomes (diabetes distress, cognition), in both T1DM and T2DM.

### Alternative model testing

An alternative model was tested, whereby diabetes distress was incorporated as an exogenous variable rather than endogenous. This model was rejected due to poor fit.

Fit indices yielded a poorer model fit compared to the initial model: χ^2^ (Chi-Square) = 172.606, *df* = 60, and χ^2^/*df* ratio = 2.877. Comparative Fit Index (CFI) = 0.913, Incremental Fit Index (IFI) = 0.915, Tucker-Lewis Index (TLI) = 0.87, Root Mean Square Error of Approximation (RMSEA) = 0.08, and Standardised Root Mean Square Residual (SRMR) = 0.091.

For the T1DM group, hypotheses 1, 2, 3, 4, 5 and 7 were non-significant and therefore unsupported. Hypothesis 6, which is part of the indirect effect was supported (β = -0.414, t = -1.96, *p* = 0.050), suggesting fatigue had a significant negative effect on resilience. Mediation analysis revealed no significant effects.

For the T2DM group, hypotheses 1, 2, 3, 4 and 7 were non-significant and therefore unsupported. Hypotheses 5 and 6, which are part of the indirect effect were supported (β = -0.059, t = -2.54, *p* = 0.011; β = -0.218, t = -3.09, *p* = 0.002, respectively), suggesting a significant effect of anxiety and fatigue on resilience. Mediation analysis revealed significant indirect effects of anxiety (-0.156, *p* = 0.001) and fatigue (-0.578, *p* = 0.001), suggesting resilience mediated the relationship between both anxiety and fatigue, and cognition.

Multigroup analysis revealed no significant differences between T1DM and T2DM groups (χ^2^ = 2.33, *p* = 0.507), suggesting the model is appropriate to both groups.

## Discussion

This study was the first to test a theoretical model whereby psychological states (anxiety/worry, fatigue) and diabetes outcomes (diabetes distress, cognition) are mediated by resilience, in both T1DM and T2DM groups. Preliminary correlations between predictor and outcome variables were as expected, based on past evidence [8, 12], in both T1DM and T2DM. All psychological state and diabetes outcome variables were significantly negatively associated with resilience, laying the groundwork for SEM. The proposed structural model achieved an acceptable model fit with no significant differences between diabetes type, suggesting the model was appropriate for both T1DM and T2DM groups.

For both T1DM and T2DM groups, direct relationships within the model were not significant and therefore not supported. However, indirect paths demonstrating the mediating effect were all significant. Bootstrapping confirmed a significant mediating effect of resilience between psychological states (anxiety, fatigue) and diabetes outcomes (diabetes distress, cognition) in both T1DM and T2DM. Additionally, the model supports an indirect relationship between diabetes distress and fatigue, which is similar to the findings of Park et al. [30], although this study looked at resilience as a mediator variable rather than diabetes symptoms. Interestingly, Lasselin et al., [41] also found data supporting higher rates of fatigue in T2DM compared to T1DM, however the SEM revealed no significant multigroup differences in fatigue.

Mediation research is necessary for advancement of psychological theory and clinical therapies [44]. These findings confirm a novel theoretical model that has the potential to optimise intervention treatments and subsequently improve diabetes self-management (i.e., improving disease prognosis and health outcomes). Resilience has also been found to play a protective role in the psychological states of other diseases, for example, protecting against: depression in adults managing cardiac disease [45]; psychological distress in cancer patients [46]; and diabetes-specific distress in diabetes patients [7]; where each study found improved health outcomes with greater resilience. Despite this, the mechanisms by which resilience acts as a protective factor are not well known [7, 45], which this study aimed to address. The mediating effect of resilience (in the context of anxiety/fatigue) can be utilised in early education interventions (e.g., conversion maps) to improve knowledge and management of diabetes outcomes [47], which can prevent serious diabetes complications (e.g., limb loss) [4]. Resilience training would provide a protective measure against negative psychological states/disorders and help improve health outcomes; this would be widely applicable to other areas of life and managing other chronic diseases.

This study has several strengths. Structural Equation Modelling allows for investigation of complex relationships simultaneously and is able to measure unobserved variables using observed variables (accounting for error measurement, rather than treating them separately) [34]. Another benefit is that SEM performs well with a range of sample sizes, including ones smaller than that of this study [e.g., 35, 36]. Yet, it is important for future studies to confirm this model using a larger diverse sample. Although this study is cross-sectional rather than longitudinal, SEM allows one to test theoretically plausible ideas about the order of variables, and thus, this study identified anxiety and fatigue as psychological risk factors that can be mediated by resilience.

This study has several areas for further investigation. Comparisons of individual difference variables such as males and females and ethnicity were not investigated, which is important for this theoretical model because findings have suggested gender and racial differences in diabetes management [42]. For example, males report more problem-focused coping methods whereas females report more negative and emotion-focused coping styles. It is important also to note total samples for T1DM and T2DM groups were mostly female (77.5% and 79.2%, respectively), which is not representative in the current diabetes literature [43]. This could suggest that females are more likely to reach out to others regarding their diabetes, which has significant implications in both healthcare and research settings. It is important to confirm these results in a more representative population regarding gender split, to ensure reliability of findings.

## Conclusions

This study showed resilience mediates the relationship between anxious and fatigued psychological states and diabetes distress and cognition in adults with T1DM and T2DM. It is recommended those devising interventions for people with T1DM and T2DM target resilience as a potential psychological mechanism; specifically, to offset problems with diabetes distress and cognition, as a consequence of anxiety/worry and fatigue. This could help improve health outcomes and quality of life in people with this lifelong condition, which in turn can positively impact mental health and wellbeing.

## Appendices

### Appendix 1: Means (and standard deviations) for participant classification information and demographics

**Table:** 

	Type 1 (*N* = 129)		Type 2 (*N* = 178)		Total (*N* = 307)
	Male (27)	Female (100)	Male (36)	Female (141)	
Age*	432.41 (178.99)		663.99 (129.65)		566.89 (190.34)
Diabetes Duration*	235.71(179.14)		97.08 (85.51)		155.16 (149.52)
HbA1c**	62.11/ 7.8 (17.01)		62.271/ 7.8 (18.68)		62.204/ 7.8 (17.93)
Ethnicity					
British/English	104		129		233
Welsh	1		0		1
Irish	3		1		4
British Asian	1		3		4
White Non-Hispanic	1		0		1
Greek	1		1		2
German	0		2		2
Mixed Caribbean	0		1		1
American	0		1		1
Greek	0		1		1
White European	0		6		6
White	16		32		48
Mixed	0		1		1

*Age and diabetes duration measured in months

**Reported dually in % and mmols/mol

### Appendix 2: Correlations (Pearson’s r) between psychological states, resilience and diabetes outcomes in Type 1 and 2 groups

**Table:** 

	PSWQ	FFS	DDS_TOT	DDS_EB	DDS_PRD	DDS_RRD	DDS_ID	DEX_TOT	DEX_VOL	DEX_INH	DEX_SR	CDRISC
PSWQ	-	0.41^**^	0.57^**^	0.59^**^	0.32^**^	0.54^**^	0.38^**^	0.44^**^	0.55^**^	0.34^**^	-0.03	-0.52^**^
FFS	0.46**	-	0.49^**^	0.49^**^	0.37^**^	0.33^**^	0.43^**^	0.30^**^	0.41^**^	0.18^*^	0.07	-0.34^**^
DDS_TOT	**0.49** ^**^	**0.50** ^**^	-	0.88^**^	0.74^**^	0.86^**^	0.81^**^	0.41^**^	0.48^**^	0.31^**^	0.15	-0.51^**^
DDS_EB	**0.53** ^**^	**0.50** ^**^	**0.89** ^**^	-	0.48^**^	0.70^**^	0.64^**^	0.43^**^	0.48^**^	0.35^**^	0.09	-0.50^**^
DDS_PRD	**0.24** ^**^	**0.32** ^**^	**0.80** ^**^	**0.60** ^**^	-	0.47^**^	0.53^**^	0.29^**^	0.29^**^	0.22^*^	0.14	-0.30^**^
DDS_RRD	**0.42** ^**^	**0.48** ^**^	**0.87** ^**^	**0.71** ^**^	**0.53** ^**^	-	0.59^**^	0.35^**^	0.43^**^	0.30^**^	0.12	-0.50^**^
DDS_ID	**0.47** ^**^	**0.39** ^**^	**0.84** ^**^	**0.71** ^**^	**0.59** ^**^	**0.65** ^**^	-	0.25^**^	0.32^**^	0.09	0.15	-0.34^**^
DEX_TOT	**0.45** ^**^	**0.46** ^**^	**0.42** ^**^	**0.43** ^**^	**0.19** ^*^	**0.44** ^**^	**0.35** ^**^	-	0.85^**^	0.83^**^	0.61^**^	-0.50^**^
DEX_VOL	**0.48** ^**^	**0.59** ^**^	**0.51** ^**^	**0.53** ^**^	**0.27** ^**^	**0.50** ^**^	**0.42** ^**^	**0.82** ^**^	-	0.58^**^	0.37^**^	-0.58^**^
DEX_INH	**0.32** ^**^	**0.31** ^**^	**0.23** ^**^	**0.25** ^**^	**0.07**	**0.29** ^**^	**0.16** ^*^	**0.82** ^**^	**0.51** ^**^	-	0.46^**^	-0.28^**^
DEX_SR	**0.13**	**0.14**	**0.18** ^*^	**0.20** ^**^	**0.10**	**0.17** ^*^	**0.15** ^*^	**0.61** ^**^	**0.34** ^**^	**0.44** ^**^	-	-0.18*
CDRISC	**-0.47** ^**^	**-0.44** ^**^	**-0.47** ^**^	**-0.46** ^**^	**-0.27** ^**^	**-0.45** ^**^	**-0.39** ^**^	**-0.41** ^**^	**-0.53** ^**^	**-0.16** ^*^	**-0.23** ^**^	-

Type 1 = top half of matrix, **Type 2 = bottom half of matrix**

Key: Psychological States: PSWQ (Measuring Anxiety); FFS (Measuring Fatigue). Resilience: CD-RISC (Measuring Resilience). Diabetes Outcomes: DDS (Measuring diabetes distress); EB (Emotional Burden); PRD (Physician-Related Distress); RRD (Regimen-Related Distress); ID (Interpersonal Distress); DEX (Measuring Cognitive dysfunction); VOL (Volition); INH (Inhibition); SR (Social Regulation)

**Correlation is significant at the 0.01 level; *Correlation is significant at the 0.05 level

### Appendix 3: Full CFA Results for Measurement Model 1, Type 1 and 2 Diabetes Groups ( T2 =  bold)

**Table:** 

Conceptual variable *(and subscales)*	Item	Factor loading	t value (C.R.)	R^2^ value	CR (composite reliability)	AVE (Average variance extracted)	Cronbach’s alpha (α)
DDS	Q1	0.79** 0.80**	Fixed **Fixed**	0.621 **0.637**	0.968 **0.977**	0.644 **0.716**	0.935 **0.951**
Emotional burden	Q14	0.93** 0.89**	12.436 **13.839**	0.873 **0.792**			
(EB)	Q8	0.87** 0.85**	11.331 **13.011**	0.760 **0.725**			
	Q3	0.87** 0.85**	11.308 **13.028**	0.758 **0.726**			
	Q11	0.70** 0.74**	8.578 **10.868**	0.495 **0.554**			
Physician related	Q4	0.81** 0.91**	Fixed **Fixed**	0.653 **0.823**			
Distress (PRD)	Q2	0.76** 0.87**	9.114 **17.405**	0.576 **0.764**			
	Q9	0.84** 0.88**	10.319 **17.555**	0.714 **0.770**			
	Q15	0.77** 0.91**	9.269 **18.951**	0.592 **0.821**			
Regimen related	Q6	0.86** 0.91**	Fixed **Fixed**	0.731 **0.828**			
Distress (RRD)	Q12	0.68** 0.85**	8.432 **16.568**	0.456 **0.731**			
	Q16	0.86** 0.78**	11.888 **13.902**	0.739 **0.615**			
	Q5	0.66** 0.60**	8.157 **9.001**	0.434 **0.355**			
	Q10	0.69** 0.90**	8.699 **18.841**	0.477 **0.817**			
Interpersonal	Q17	0.87** 0.88**	Fixed **Fixed**	0.754 **0.768**			
Distress (IP)	Q7	0.77** 0.87**	10.197 **14.972**	0.600 **0.749**			
	Q13	0.85** 0.84**	11.595 **14.348**	0.724 **0.711**			
PSWQ	Q5	0.898** 0.911**	Fixed **Fixed**	0.807 **0.831**	0.971 **0.980**	0.699 **0.769**	0.971 **0.980**
(Q8 removed)	Q7	0.869** 0.929**	14.875 **21.816**	0.756 **0.862**			
	Q4	0.893** 0.909**	15.860 **20.461**	0.797 **0.827**			
	Q15	0.897** 0.909**	16.073 **20.459**	0.805 **0.827**			
	Q14	0.882** 0.911**	15.400 **20.589**	0.778 **0.830**			
	Q3	0.849** 0.906**	14.077 **20.246**	0.720 **0.821**			
	Q13	0.877** 0.897**	15.162 **19.691**	0.768 **0.805**			
	Q10	0.859** 0.891**	14.457 **19.320**	0.737 **0.793**			
	Q11	0.835** 0.896**	13.590 **19.659**	0.697 **0.804**			
	Q2	0.846** 0.883**	13.976 **18.879**	0.715 **0.780**			
	Q6	0.829** 0.873**	13.398 **18.337**	0.688 **0.762**			
	Q16	0.771** 0.856**	11.630 **17.465**	0.594 **0.733**			
	Q9	0.765** 0.789**	11.459 **14.628**	0.585 **0.622**			
	Q12	0.727** 0.802**	10.509 **15.133**	0.529 **0.644**			
	Q1	0.715** 0.772**	10.205 **14.020**	0.511 **0.596**			
FFS	Q1	0.902** 0.908**	Fixed **Fixed**	0.814 **0.825**	0.925 **0.909**	0.673 **0.632**	0.916 **0.899**
(Q7 removed)	Q2	0.883** 0.911**	14.786 **18.916**	0.779 **0.829**			
	Q6	0.792** 0.838**	11.877 **15.742**	0.628 **0.702**			
	Q4	0.828** 0.764**	12.925 **13.188**	0.685 **0.584**			
	Q3	0.836** 0.762**	13.175 **13.103**	0.699 **0.580**			
	Q5	0.659** 0.521**	8.773 **7.527**	0.434 **0.271**			
DEX (3 factors)	Q8	0.706** 0.722**	Fixed **Fixed**	0.498 **0.521**	0.915 **0.919**	0.477 **0.489**	0.881 **0.882**
DEX: Volition	Q19	0.756** 0.746**	7.641 **9.285**	0.571 **0.557**			
	Q10	0.627** 0.778**	6.437 **9.669**	0.393 **0.606**			
	Q4	0.704** 0.713**	7.174 **8.888**	0.496 **0.509**			
	Q18	0.748** 0.776**	7.578 **9.646**	0.560 **0.603**			
DEX: Inhibition	Q9	0.666** 0.687**	Fixed **Fixed**	0.444 **0.471**			
	Q2	0.691** 0.544**	6.654 **6.477**	0.477 **0.296**			
	Q17	0.707** 0.813**	6.779 **9.146**	0.499 **0.661**			
	Q16	0.735** 0.632**	6.997 **7.431**	0.541 **0.400**			
	Q15	0.610** 0.615**	5.989 **7.247**	0.372 **0.378**			
DEX: Social	Q20	0.763** 0.682**	Fixed **Fixed**	0.582 **0.465**			
Regulation	Q13	0.535** 0.639**	3.892 **5.112**	0.286 **0.408**			
CD-RISC:	Q5	0.808** 0.756**	Fixed Fixed	0.652 0.572	0.955 **0.950**	0.504 **0.477**	0.954 **0.949**
Q9,18,20,3 removed	Q17	0.805** 0.744**	10.644 **10.380**	0.648 **0.554**			
	Q4	0.788** 0.761**	10.329 **10.659**	0.621 **0.580**			
	Q11	0.809** 0.734**	10.723 **10.214**	0.654 **0.538**			
	Q23	0.704** 0.787**	8.877 **11.076**	0.495 **0.619**			
	Q22	0.821** 0.696**	10.957 **9.619**	0.674 **0.485**			
	Q7	0.770** 0.723**	10.009 **10.049**	0.593 **0.523**			
	Q21	0.756** 0.719**	9.770 **9.985**	0.572 **0.518**			
	Q24	0.730** 0.741**	9.310 **10.322**	0.533 **0.548**			
	Q14	0.710** 0.737**	8.977 **10.266**	0.504 **0.543**			
	Q8	0.710** 0.699**	8.973 **9.668**	0.503 **0.489**			
	Q12	0.666** 0.728**	8.274 **10.122**	0.443 **0.530**			
	Q25	0.682** 0.706**	8.528 **9.772**	0.465 **0.498**			
	Q16	0.711** 0.641**	8.995 **8.763**	0.505 **0.410**			
	Q19	0.751** 0.613**	9.679 **8.353**	0.564 **0.376**			
	Q15	0.707** 0.634**	8.927 **8.669**	0.499 **0.402**			
	Q6	0.613** 0.636**	7.472 **8.694**	0.376 **0.405**			
	Q1	0.654** 0.607**	8.086 **8.256**	0.427 **0.368**			
	Q13	0.549** 0.628**	6.553 **8.574**	0.301 **0.395**			
	Q10	0.530** 0.613**	6.295 **8.350**	0.281 **0.376**			
	Q2	0.527** 0.551**	6.251 **7.429**	0.277 **0.303**			

**p < 0.001, T1 *n* = 129, **T2 *n* = 178**

### Appendix 4: Bootstrapping Mediation Analysis in Type 1 Diabetes Group

To confirm the presence of mediation, bootstrapping was used to calculate direct and indirect effects in type 1 diabetes (see below).

**Table:** 

Mediation analysis in type 1 diabetes			
Hypothesis	Direct Effect	Indirect Effect	Result
Anxiety → Res → D.Distress	− 0.018 (ns)	0.086**	Sig Mediation
Anxiety → Res → Cognition	− 0.007 (ns)	0.069**	Sig Mediation
Fatigue → Res → D.Distress	n/a	0.299**	Sig Mediation
Fatigue → Res → Cognition	0.023 (ns)	0.240**	Sig Mediation

This table confirms a mediating effect of resilience on the relationships between mood states (anxiety, fatigue) and diabetes outcomes (diabetes distress, cognition) in type 1 diabetes

***p < 0.001; **p <  0.05

### Appendix 5: Bootstrapping mediation analysis in Type 2 diabetes group

To confirm the presence of mediation, bootstrapping was used to calculate direct and indirect effects in type 2 diabetes (see below).

**Table:** 

Mediation analysis in type 2 diabetes			
Hypothesis	Direct Effect	Indirect Effect	Result
Anxiety → Res → D.Distress	− 0.001 (ns)	0.059**	Sig Mediation
Anxiety → Res → Cognition	0.008 (ns)	0.061**	Sig Mediation
Fatigue → Res → D.Distress	n/a	0.201**	Sig Mediation
Fatigue → Res → Cognition	0.026 (ns)	0.209**	Sig Mediation

This table confirms a mediating effect of resilience on the relationships between mood states (anxiety, fatigue) and diabetes outcomes (diabetes distress, cognition) in type 2 diabetes

***p < 0.001;**p < 0.05

### Appendix 6: Fit Statistics of alternative model comparisons for Type 1 and 2 diabetes

**Table:** 

Model	χ^2^	df	∆ χ^2^	∆df	χ^2^/df	CFI	RMSEA	SRMR
Base model (see Fig. 1)	*139.905*	*58*	*–*	*–*	*2.41*	0.94	0.07	0.07
Model 1	*142.843*	*60*	2.938**	*2*	*2.38*	0.94	0.07	0.07
Model 2	*141.464*	*60*	1.559**	*2*	*2.34*	0.94	0.07	0.07
Model 3	*141.580*	*60*	1.675**	*2*	*2.36*	0.94	0.07	0.07
Model 4	*146.908*	*64*	7.003**	*6*	*2.30*	0.94	0.07	0.07

**p<0.001; *p.<0.05

Model 1: The path of Hypothesis 1 (**H1** Anxiety/Worry → Diabetes Distress) was constrained to zero.

Model 2: The path of Hypothesis 3 (**H2** Anxiety/Worry → Cognition) was constrained to zero.

Model 3: The path of Hypothesis 4 (**H3** Fatigue → Cognition) was constrained to zero.

Model 4: The paths of hypotheses 1,2 & 3 were constrained to zero.

## Data Availability

The datasets generated and analysed during the current study are available from the corresponding author on reasonable request.
